# Analysis of Soccer Players’ Positional Variability During the 2012 UEFA European Championship: A Case Study

**DOI:** 10.1515/hukin-2015-0078

**Published:** 2015-10-14

**Authors:** Felipe Arruda Moura, Juliana Exel Santana, Nathália Arnosti Vieira, Paulo Roberto Pereira Santiago, Sergio Augusto Cunha

**Affiliations:** 1 Laboratory of Applied Biomechanics, Sport Sciences Department, State University of Londrina, Londrina, Brazil.; 2 Laboratory of Instrumentation for Biomechanics, College of Physical Education, University of Campinas, Campinas, Brazil.; 3 Biomechanics and Motor Control Laboratory, School of Physical Education and Sport of Ribeirão Preto, University of São Paulo, Ribeirão Preto, Brazil.

**Keywords:** match analysis, heat map, principal component analysis, tactics

## Abstract

The purpose of this study was to analyse players’ positional variability during the 2012 UEFA European Championship by applying principal component analysis (PCA) to data gathered from heat maps posted on the UEFA website. We analysed the teams that reached the finals and semi-finals of the competition. The players’ 2D coordinates from each match were obtained by applying an image-processing algorithm to the heat maps. With all the players’ 2D coordinates for each match, we applied PCA to identify the directions of greatest variability. Then, two orthogonal segments were centred on each player’s mean position for all matches. The segments’ directions were driven by the eigenvectors of the PCA, and the length of each segment was defined as one standard deviation around the mean. Finally, an ellipse was circumscribed around both segments. To represent player variability, segment lengths and elliptical areas were analysed. The results demonstrate that Portugal exhibited the lowest variability, followed by Germany, Spain and Italy. Additionally, a graphical representation of every player’s ellipse provided insight into the teams’ organisational features throughout the competition. The presented study provides important information regarding soccer teams’ tactical strategy in high-level championships that allows coaches to better control team organisation on the pitch.

## Introduction

Researchers in sports science, particularly those who study team sports, have attempted to yield quantitative data about technical, physical and tactical aspects of performance during competitive situations. The technical elements of player’s performance in soccer, such as control, passing, a shot-to-goal ratio and fouls, are measured by notational systems and have been widely studied to identify patterns of play (Barros et al., 2006; Bate, 1988; Hughes and Franks, 2005; Yamanaka et al., 1993). Video-based systems can also automatically and accurately gather players’ positional data to obtain the distances they cover and their velocities for use as indicators of an athletes’ physical condition (Barros et al., 2007; Bradley et al., 2009; Di Salvo et al., 2007; Di Salvo et al., 2009).

Positional data have also been used extensively to study tactical issues in team sports such as basketball (Bourbousson et al., 2010), futsal (Moura et al., 2011) and soccer (Moura et al., 2013; Moura et al., 2012; Okihara et al., 2004; Yue et al., 2008). These studies present information about team organisation and tactical dynamics during matches. Furthermore, tactics in team sports have been evaluated using a dynamical systems approach (Bourbousson et al., 2010; Gréhaigne et al., 1997; McGarry et al., 2002). Dyads of players and/or teams have been considered in these investigations to analyse a team’s organisational level during attack plays that precede goals (Gréhaigne et al., 1997).

However, from players’ trajectories, it is also possible to analyse movement variability during matches. Such variability has been studied to understand the main factors and constraints that affect players’ actions. Considering the tactical behaviour of players, the variability of players’ trajectories can be viewed as an interesting indicator in characterising soccer players within their specific tactical zones (Couceiro et al., 2014). The study of individual movement variability can explain how players performance in complex and dynamic environments, such as soccer matches, can manage space and time as a function of specific constraints (Davids et al., 2005; Silva et al., 2014).

From players trajectories obtained using GPS devices during small-sided games, a recent study (Silva et al., 2014) evaluated players’ movement variability as a function of changes in field dimension and compared the behaviour of players of two different levels (national and regional). The authors’ presented linear (percentage of coefficient variation) and nonlinear (Shannon and sample entropies) analyses to examine the variability of the players’ spatial-temporal characteristics. The main result indicated that increases in pitch size resulted in more restricted action zones for both groups. National players were more sensitive to pitch modifications and displayed more variability than regional players did in small and intermediate pitches.

In another recent investigation (Couceiro et al., 2014), the concept of stability for soccer was presented as not only the resistance to a perturbation, but also a player’s capability to maintain his trajectory within a specific region (such as his tactical position on the field). The existence of a stable equilibrium point implies the existence of a “restoring force” that is directed towards the equilibrium point. Thus, players usually converge at an equilibrium point that is defined by their tactical position. Using entropy measures from players’ trajectories, the authors presented stability differences among soccer positions that may be related to their specific tactical missions.

Furthermore, a previous study applied principal component analysis (PCA) to players’ positional data to understand tactical performance in soccer (Barros et al., 2006). PCA is a multivariate statistical method of data analysis that transforms a set of observations of different variables into a set of uncorrelated variables, called principal components. The principal components are linear combinations of the original variables and are orthogonal to each other. The observations are then projected onto each principal component, yielding a new variable of which variance is the highest among all possible choices for each component (Jolliffe, 2002). Barros and colleagues (2006) analysed players’ positional variability on the pitch, based on players’ planar coordinates when performing a technical action, by applying PCA. The authors graphically represented the two principal components related to 2D positional data and indicated the directions on the pitch where players performed their technical actions.

Despite the importance of these studies and results, they depend on players’ 2D planar coordinate data to investigate positional variability during a match. However, it is possible that coaches do not have access to such technology to obtain players’ trajectories data. Additionally, coaches may need to investigate adversary tactical behaviour, such as players’ positional variability, previous to a match, but clearly they cannot collect such information. As an alternative solution, advances in data collection technologies for team sports have allowed for access to performance indicators online during matches. In 2012, the UEFA European Championship official website provided several match statistics as well as information about team and player performances. One of these performance indicators was the heat map of each player, which indicates the frequency of a player’s position at a given location on the pitch. Thus, the region where a player visited most may represent the equilibrium point that is defined by his tactical position (Couceiro et al., 2014). Although the heat maps do not provide player coordinates as a function of time to allow for the quantification of positional variability during a match, using image processing it is possible to detect the pitch location that a given player visited most. The same procedure can be performed during a given championship, and player positional variability throughout the matches can be described. Furthermore, the player heat maps are calculated and presented on an individual basis and, consequently, do not provide collective information about how a team was organised during a match or how this organisation evolved during the championship. The representation of team data can be helpful information for coaches to obtain knowledge on regarding team tactics and can facilitate the analysis of collective behaviour changes during each match. In this sense, PCA applied to heat maps over the course of a championship can provide the overall directions of highest variability for each player and yield a portrait of team tactics throughout an entire competition. Therefore, the aim of this study was to propose an analysis of players’ positional variability during the 2012 UEFA European Championship (EURO 2012) by applying PCA to the data provided by heat maps posted on the UEFA official website.

## Material and Methods

We analysed the heat maps of the starting players for the teams from Spain, Italy, Germany and Portugal during the EURO 2012. Once Spain and Italy reached the finals, we analysed their six matches, whereas Portugal and Germany only had five matches that could be analysed.

**Figure 1:**
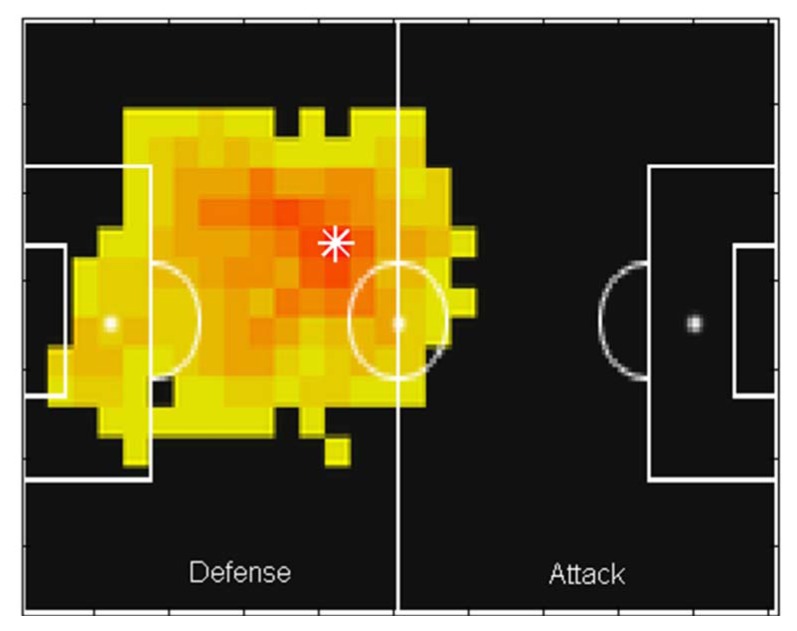
Example of the treatment of heat map images and the identification of a 2D player position for further PCA application.

The heat map figures of the players for each match were publically available on the UEFA official website (www.uefa.com) throughout the course of the competition. A player’s heat map is a 2D shaded-surface plot that represents the locations on the pitch that the player “visited” most frequently. Usually, this information is obtained by video-based tracking methods that provide a player’s position as a function of time. On a heat map, the pitch locations the player covered most frequently are coloured red, whereas the locations never frequented by the player are coloured black (Figure 1).

To identify a player’s main position during a match, based on his heat map, a Matlab® algorithm was created to automatically recognise red pixels in the figure. Thus, each image was imported into the Matlab® software as an array with dimensions of 135 × 202 × 3. The first and second elements represented the pixel resolution of the heat maps, and the third element represented the red, green and blue intensities of each pixel. The figure was imported as an 8-bit RGB image in which the colour components were represented as integers in the range [0, 255]. In the algorithm employed, the red pixels were recognised using a filter to identify integers greater than 240 for the red array, lower than 35 for the blue array and lower than 10 for the green array (see appendix). After obtaining the 2D coordinates that represented the red pixels, we calculated the mean coordinate values that represented player positions in each match (indicated by a white asterisk in Figure 1). Finally, these mean coordinates were converted from pixels to metres based on the dimensions of a standard football pitch, i.e., 110 × 75 m.

**Figure 2:**
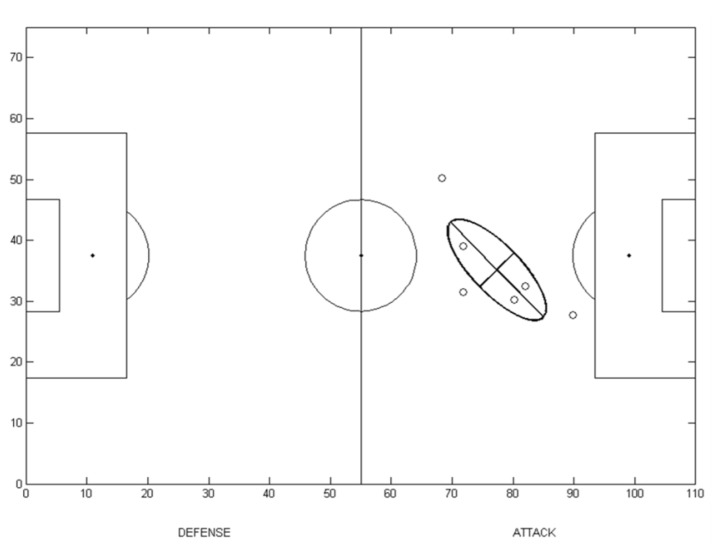
Graphical representation of player’s position variability resulting from PCA. The small circles represent the player’s mean position obtained from each of six matches. The black line segments inside the ellipse represent the variability associated with the first and second principal components.

Principal component analysis was applied to identify player position variability during the competition, using each player’s mean position on the pitch (see appendix). The data set consisted of one matrix for each player R(x(i), y(i)), where x(i) and y(i) represent the mean coordinates on the pitch during the match, with i=1,…,N, where N is the total number of matches (N = 6 for Spain and Italy and N = 5 for Germany and Portugal). We applied PCA to the covariance matrix of the R input data set, and the following variables were obtained: a) the principal components of the new coordinate system, the eigenvectors u and v, and b) the eigenvalues (λ1 and λ2) corresponding to the total variability explained by each principal component. With these variables, a graphical representation was created for each player. Two orthogonal segments were centred on the mean position of each player for all matches (i.e., the mean values of R). The segments’ directions were driven by the eigenvectors of the PCA, and the length of each segment was defined as one standard deviation (σ1 and σ2) around the mean, which was calculated by extracting the square root of each eigenvalue, λ1 and λ2. Figure 2 presents a graphical representation of the orthogonal segments for one player. Each small circle represents the player’s mean coordinates during each match.

Finally, to quantitatively analyse player position variability during the competition, three variables were registered: elliptical area (given by the expression A=π*σ1*σ2), which represents each player’s general variability, first component length (σ1) and second component length (σ2), which represent the amount of variability in the two directions that varied the most. Through descriptive analysis, the values of these variables obtained for each team and for each position group were expressed by the median and interquartile range calculated according to McGill et al. (1978).

## Results

**Figure 3:**
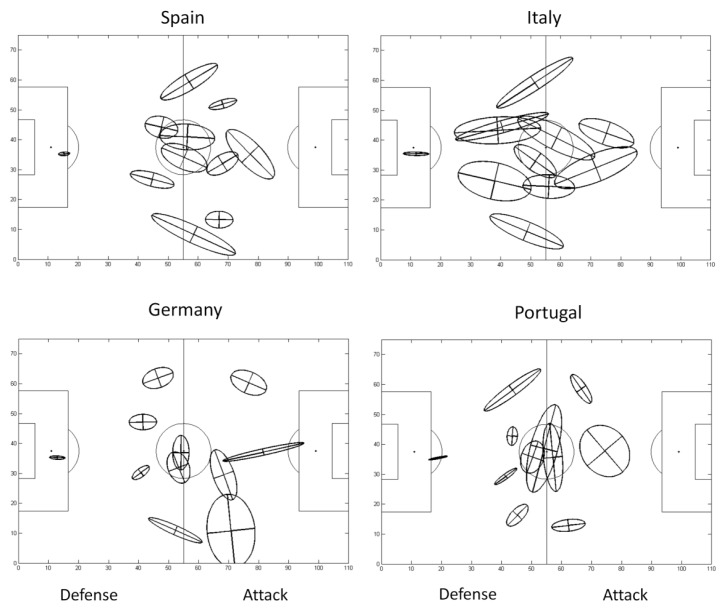
PCA calculated for each of the 11 players on the teams from Spain, Italy, Germany and Portugal during EURO 2012.

Figure 3 presents the PCA results obtained for the players from Spain, Italy, Germany and Portugal during all EURO 2012 matches. The figure depicts each team’s organisation on the pitch during the competition, as well as all players’ positional variability. The largest individual elliptical areas and their orthogonal segments represent major changes to the teams’ organisational patterns. As a result, it is possible to visually confirm that Italy implemented significant changes in its players’ positions as PCA of the data resulted in the largest ellipses and orthogonal segments. By contrast, small ellipses can be observed for the other teams, representing reduced variability during the competition.

**Figure 4:**
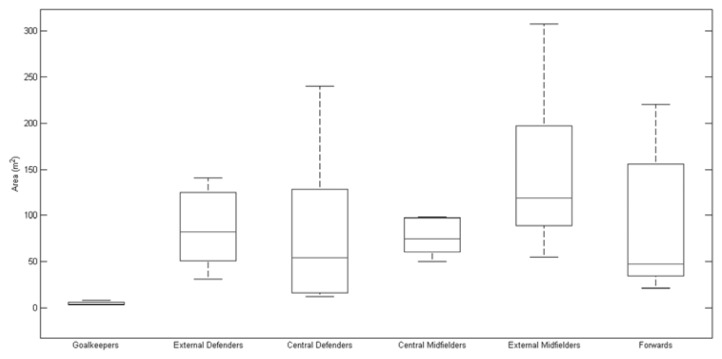
Players’ position variability determined for the finalist teams of EURO 2012, represented by the elliptical areas obtained by PCA.

**Figure 5:**
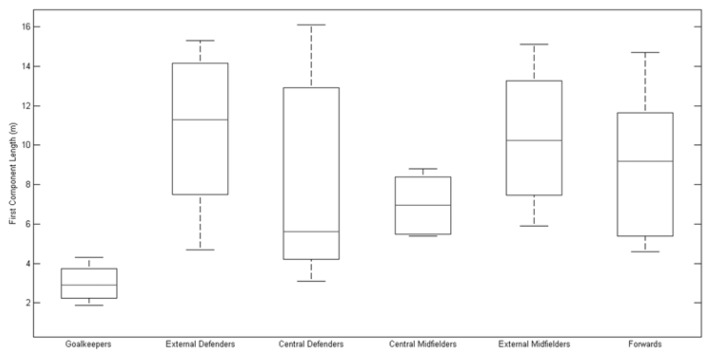
Players’ position variability determined for the finalist teams of EURO 2012, represented by the first component length obtained by PCA.

**Figure 6:**
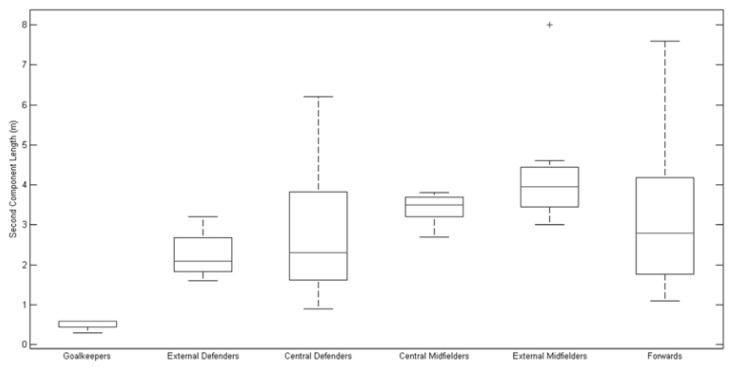
Players’ position variability determined for the finalist teams of EURO 2012, represented by the second component length obtained by PCA.

To better comprehend the teams’ variability in a player position during the competition, Table 1 presents the median and the interquartile ranges of all variables for each team analysed and the values obtained for each player. The quantitative results confirm that Italy, which exhibited the largest elliptical areas and length values for the components, did in fact demonstrate the highest variability among all the teams analysed. Furthermore, Portugal, which exhibited the smallest elliptical areas and first and second segment lengths, demonstrated the lowest variability. Additionally, Figures 4, 5 and 6 present boxplots that represent players’ variability according to a playing position. The results show that the external midfielders and external defenders presented the greatest variability throughout the championship compared with that observed for the other positions, mainly due to the elliptical area and first component length (Figures 4 and 5, respectively).

## Discussion

The aim of this study was to analyse players’ positional variability during the 2012 UEFA European Championship (EURO 2012) by applying PCA to heat maps to evaluate the teams’ tactical behaviour patterns throughout the competition. Specifically, we analysed the teams that reached the competition’s final and semi-final rounds. Portugal presented the lowest variability, followed by, in ascending order, Germany, Spain and Italy. External midfielders and external defenders presented the greatest variability among all positions.

The analysis of the results must be interpreted carefully because variability has been studied from different viewpoints. From a traditional motor learning perspective, variability has been considered to be an essential element of the healthy function of a system under investigation and consequently offers flexibility in adapting to perturbations (Hamill et al., 1999; van Emmerik and van Wegen, 2000; Wilson et al., 2008). When addressing movement coordination from a dynamical systems perspective, measures of variability become critical to understanding movement dynamics (Hamill et al., 2000). Specifically to soccer, players’ positional variability characterises the tactical zones where they play during matches and competition (Couceiro et al., 2014) and may allow for the stratification of the behaviour of players from different skill levels (Silva et al., 2014).

Thus, it is essential in such an analysis that the role of variability within the system is identified, but it is difficult to determine whether this variability is beneficial. In the current study, the variability of parameters related to tactical behaviour in soccer presents advantages and disadvantages. Low variability could indicate pattern maintenance by a team throughout a match or competition. Teams with small principal component lengths and elliptical areas could be characterised as systems not only with a high level of organisation but also with predictable behaviour, which could facilitate a precise evaluation of their performance by their opponents. High variability could signify a team’s difficulty in maintaining a set formation. On the other hand, significant changes in a team’s structure or behaviour could yield unpredictability or a higher capacity of adaptation to unexpected situations during a match or championship. From a dynamical systems perspective, variability is not inherently good or bad, but rather reflects the variety of patterns used to complete a task (Haken et al., 1985), suggesting that the system is flexible enough to search for the optimal solution (Miller et al., 2010).

The manner in which principal components and their elliptical areas are distributed on the pitch can also reveal interesting visual features about tactical patterns. The Spanish and German players, for example, exhibited ellipses that were more distant from each other and that had few intersections between them. Such an outcome may indicate that the players’ positions were well defined throughout the championship, in stark contrast to those of players from Italy and Portugal. Both Italy and Portugal, in this case, displayed ellipses that overlapped regularly, which could mean that their players changed playing positions at some point or even that they played in the same position as other teammates during the competition. This type of information could be quite helpful to coaches and assistants who need to determine whether the tactics adopted or proposed were executed by their players as a training control parameter.

Our results also provided the positional variability discriminated by a player position. Although previous studies (Barros et al., 2006; Couceiro et al., 2014) had reported variability analyses based on players’ positions, to the best of our knowledge, this study was the first that presented an analysis of positional variability throughout a championship. Our findings indicate that external midfielders and external defenders exhibited the greatest variability, corroborating the study of Barros and colleagues (Barros et al., 2006). Indeed, during a match, each player has a specific tactical mission and an intervention region that provides some organisation to a team’s collective dynamics. Although each player moves to support the defensive and offensive phases, the player eventually returns to his main tactical region due to his positional role (Couceiro et al., 2014). This behaviour becomes very clear when each match is submitted to variability analysis, as presented by Couceiro and colleagues using players’ planar coordinates and heat maps (Couceiro et al., 2014). However, when we consider players’ behaviour over the course of an entire championship, the tendency to return to the same region, for the external midfielders and external defenders, for example, is not as distinct. This outcome may be related to these players’ tactical roles. Once the other positions have well-defined functions (i.e., only to attack or to defend) during a match, the external defenders, for example, not only usually adopt a defensive role but also support the midfielders during attack phases (Couceiro et al., 2014). Thus, it is possible that during the championship, for some matches, these players adopted a more defensive role, whereas in other matches, they adopted a more offensive role, therefore explaining their variability.

Similarly, it is important to interpret an ellipse’s shape, as shown graphically by Figure 3. An ellipse’s shape is defined by its component lengths. This parameter is related to the eccentricity of the area covered tactically by a player and is related to the ratio of the variances associated with the first and second components (Barros et al., 2006). In other words, a more “stretched” ellipse represents greater variability in one direction, whereas a more “circular” ellipse represents proportional variability in both directions. For example, a previous study (Barros et al., 2006) reported that external defenders presented lower eccentricity than players in other positions due to their main role, which essentially is to move in one direction during attacking and defending actions. This behaviour also explains why players’ trajectories are more unpredictable for one direction than for another (Couceiro et al., 2014). Thus, the ellipses of Figure 3 represent the magnitude of the positional variability and also provide information regarding how this variability occurred.

The present study employed an innovative approach using broadcast data provided online during matches to quantify tactical issues in soccer. By applying an image-processing algorithm to heat maps published online by UEFA during EURO 2012, we could calculate the variability of players’ tactical patterns using PCA applied to each player’s position. PCA is a statistical method used to reduce data dimensions to represent them as components that contain the greatest amount of data set variability (Barros et al., 2006; Daffertshofer et al., 2004). However, in this study, we did not reduce the data dimensions. Instead, we used the components to represent player position variability throughout the competition. One of the advantages of this particular method is that the information describing the variability of each individual data set is maintained. Thus, the variability of a player’s position is not represented in just one external, unique and predefined coordinate system, such as the field coordinate system. The data sets generate a coordinate system from their own distribution (Barros et al., 2006). This new coordinate system is associated with the percentage of total variability explained by both principal components.

From these parameters, coaches can obtain the directions of highest variability and associate them with their players’ positions. The other notable benefit of the presented method is its graphical representation of positional data. This representation is relevant to practical applications as, in addition to pattern variability, it can be related to a team’s organisation on the pitch. Additionally, it may yield intuitive ideas about the tactical systems predominantly adopted. Although we have analysed team behaviour without considering opponents’ tactical organisation, further investigations should be performed to analyse positional variability considering the interaction between teams and their adversaries. Thus, the features of space-sharing between teammates and opponents may explain why some players present greater variability than others during a given match or competition.

The presented method is a viable tool that provides coaches with important information regarding soccer team’s tactical aspects during matches and even over the course of an extended competition. This tool can also be useful to coaches and assistants in preparing for future competitions as a method for evaluating and controlling their own team’s tactical behaviour as well as understanding that of their opponents.

## Figures and Tables

**Table 1:** Players’ position variability determined for the finalist teams of EURO 2012, represented by the median (interquartile range) of elliptical areas and first and second eigenvalue lengths obtained by PCA

Teams	Players	Ellipsis Area (m^2^)	Variables First Component Length (m)	Second Component Length (m)
Spain	Goalkeeper	3.6	1.9	0.6
	External defender	141.0	15.3	2.9
	External defender	95.9	11.0	2.8
	Central Defender	54.2	7.5	2.3
	Central Defender	62.4	5.6	3.6
	Central Midfielder	97.9	8.1	3.8
	External Midfielder	54.6	5.9	3.0
	External Midfielder	124.0	9.1	4.3
	Forward	40.5	4.6	2.8
	Forward	136.1	10.9	4.0
	Forward	21.1	4.8	1.4
	**Median (IQR)**	**62.4 (73.5)**	**7.5 (5.5)**	**2.9 (1.3)**

Italy	Goalkeeper	7.8	4.3	0.6
	External defender	132.8	13.2	3.2
	External defender	117.4	15.1	2.5
	Central Defender	240.2	12.4	6.2
	Central Defender	103.8	16.1	2.1
	Central Defender	202.9	14.4	4.5
	Central Midfielder	84.4	8.4	3.2
	External Midfielder	106.5	8.6	3.9
	External Midfielder	177.9	14.2	4.0
	Forward	215.5	14.7	4.7
	Forward	118.6	9.7	3.9
	**Median (IQR)**	**118.6 (92.2)**	**13.2 (5.7)**	**3.9 (1.7)**

Germany	Goalkeeper	4.7	2.6	0.6
	External defender	47.5	9.7	1.6
	External defender	54.0	5.3	3.2
	Central Defender	15.4	3.4	1.4
	Central Defender	37.4	4.6	2.6
	Central Midfielder	60.6	5.4	3.5
	Central Midfielder	49.7	5.8	2.7
	Central Midfielder	97.2	8.8	3.5
	External Midfielder	307.8	12.3	8.0
	External Midfielder	72.5	6.3	3.7
	Forward	47.0	13.8	1.1
	**Median (IQR)**	**49.7 (29.7)**	**5.8 (4.8)**	**2.7 (2.1)**

Portugal	Goalkeeper	2.9	3.2	0.3
	External defender	30.9	4.7	2.1
	External defender	69.3	11.6	1.9
	Central Defender	17.0	3.1	1.7
	Central Defender	12.4	4.5	0.9
	Central Midfielder	65.2	5.5	3.7
	External Midfielder	216.0	15.1	4.6
	External Midfielder	113.2	11.4	3.2
	Forward	34.6	5.6	2.0
	Forward	220.8	9.2	7.6
	Forward	33.5	5.7	1.9
	**Median (IQR)**	**34.5 (81.8)**	**5.6 (6.3)**	**2.0 (1.8)**

## Appendix

- Essentially, two algorithms were created for data collection. The first one was created to recognise the red pixels in each figure and to calculate the mean pixel coordinates using the following commands:
- data=imread(figure_file); % This procedure imports the figure file as a matrix data
- [lines, columns]=find(data(:,:,1)>240 & data(:,:,2)<35 & data(:,:,3)<10); % Identify the lines and columns of the “data_double” matrix that represent the red pixels.
- Mean_coordinates = [mean(columns) mean(lines)]; % Calculate the mean pixel coordinates
- After storing the player coordinates during the matches in matrix **R**(*x(i)*, *y(i)*), where *x(i)*, *y*(*i*) are the mean coordinates on the pitch during each match, with *i*=1,…,*N*, where *N* is the total number of matches (*N* = 6 for Spain and Italy and *N* = 5 for Germany and Portugal), we calculated the eigenvalues and eigenvectors using the “princomp.m” Matlab^®^ algorithm to provide information regarding player variability as follows:
- [eigenvectors, scores, eigenvalues]=princomp(R_matrix);

## References

[b1-jhk-47-225] Barros RML, Cunha SA, Magalhães WJ, Guimarães MF (2006). Representation and analysis of soccer players’ actions using principal components. J Hum Movement Stud.

[b2-jhk-47-225] Barros RML, Misuta MS, Menezes RP, Figueroa PJ, Moura FA, Cunha SA, Anido R, Leite NJ (2007). Analysis of the distances covered by first division Brazilian soccer players obtained with an automatic tracking method. J Sports Sci Med.

[b3-jhk-47-225] Bate R (1988). Football chance: tactics and strategy. Science and Football.

[b4-jhk-47-225] Bourbousson J, Seve C, McGarry T (2010). Space-time coordination dynamics in basketball: Part 1. Intra- and inter-couplings among player dyads. J Sports Sci.

[b5-jhk-47-225] Bradley PS, Sheldon W, Wooster B, Olsen P, Boanas P, Krustrup P (2009). High-intensity running in English FA Premier League soccer matches. J Sports Sci.

[b6-jhk-47-225] Couceiro MS, Clemente FM, Martins FML, Machado JAT (2014). Dynamical stability and predictability of football players: the study of one match. Entropy.

[b7-jhk-47-225] Daffertshofer A, Lamoth CJC, Meijer OG, Beek PJ (2004). PCA in studying coordination and variability: a tutorial. Clinical Biomechanics.

[b8-jhk-47-225] Davids K, Araújo D, Shuttleworth R, iRT, CJ, Araújo D (2005). Science & Football V.

[b9-jhk-47-225] Di Salvo V, Baron R, Tschan H, Calderon Montero FJ, Bachl N, Pigozzi F (2007). Performance characteristics according to playing position in elite soccer. Int J Sports Med.

[b10-jhk-47-225] Di Salvo V, Gregson W, Atkinson G, Tordoff P, Drust B (2009). Analysis of high intensity activity in Premier League soccer. Int J Sports Med.

[b11-jhk-47-225] Gréhaigne JF, Bouthier D, David B (1997). Dynamic-system analysis of opponent relationships in collective actions in soccer. J Sports Sci.

[b12-jhk-47-225] Haken H, Kelso JAS, Bunz H (1985). A Theoretical-Model of Phase-Transitions in Human Hand Movements. Biological Cybernetics.

[b13-jhk-47-225] Hamill J, Haddad JM, McDermott WJ (2000). Issues in quantifying variability from a dynamical systems perspective. Journal of Applied Biomechanics.

[b14-jhk-47-225] Hamill J, van Emmerik REA, Heiderscheit BC, Li L (1999). A dynamical systems approach to lower extremity running injuries. Clinical Biomechanics.

[b15-jhk-47-225] Hughes M, Franks I (2005). Analysis of passing sequences, shots and goals in soccer. J Sports Sci.

[b16-jhk-47-225] Jolliffe IT (2002). Principal component analysis.

[b17-jhk-47-225] McGarry T, Anderson DI, Wallace SA, Hughes MD, Franks IM (2002). Sport competition as a dynamical self-organizing system. J Sports Sci.

[b18-jhk-47-225] McGill R, Tukey JW, Larsen WA (1978). Variations of box plots. Am Stat.

[b19-jhk-47-225] Miller RH, Chang R, Baird JL, Van Emmerik REA, Hamill J (2010). Variability in kinematic coupling assessed by vector coding and continuous relative phase. Journal of biomechanics.

[b20-jhk-47-225] Moura FA, Martins LE, Anido RO, Ruffino PR, Barros RM, Cunha SA (2013). A spectral analysis of team dynamics and tactics in Brazilian football. J Sports Sci.

[b21-jhk-47-225] Moura FA, Martins LEB, Anido RO, Barros RML, Cunha SA (2012). Quantitative analysis of Brazilian football players’ organisation on the pitch. Sports Biomech.

[b22-jhk-47-225] Moura FA, Santana JE, Marche AL, Aguiar TH, Rodrigues ACMA, Barros RML, Cunha SA (2011). Quantitative analysis of futsal players’ organization on the court. Rev Port Cien Desp.

[b23-jhk-47-225] Okihara K, Kan A, Shiokawa M, Choi CS, Deguchi T, Matsumoto M, Higashikawa Y (2004). Compactness as a strategy in a soccer match in relation to a change in offence and defense. J Sports Sci.

[b24-jhk-47-225] Silva P, Aguiar P, Duarte R, Davids K, Araújo D, Garganta J (2014). Effects of pitch size and skill level on tactical behaviours of Association Football players during small-sided and conditioned games. International Journal of Sports Science and Coaching.

[b25-jhk-47-225] van Emmerik REA, van Wegen EEH (2000). Symposium on variability and stability in human movement. Journal of Applied Biomechanics.

[b26-jhk-47-225] Wilson C, Simpson SE, Van Emmerik REA, Hamill J (2008). Coordination variability and skill development in expert triple jumpers. Sports Biomech.

[b27-jhk-47-225] Yamanaka K, Hughes M, Lott M (1993). An analysis of playing patterns in the 1990 World Cup for Association Football. Science and Football II.

[b28-jhk-47-225] Yue Z, Broich H, Seifriz F, Mester J (2008). Mathematical Analysis of a Soccer Game. Part I: Individual and Collective Behaviors. Stud Appl Math.

